# Antibacterial Efficacy of Cold Atmospheric Plasma, Photodynamic Therapy with Two Photosensitizers, and Diode Laser on Primary Mandibular Second Molar Root Canals Infected with *Enterococcus faecalis*: An In Vitro Study

**DOI:** 10.1155/2023/5514829

**Published:** 2023-04-21

**Authors:** Leyli Haghighi, Arash Azizi, Mehdi Vatanpour, Gholamhossein Ramezani

**Affiliations:** ^1^Department of Pediatric Dentistry, Tehran Dental Branch, Islamic Azad University, Tehran 19395/1495, Iran; ^2^Department of Oral and Maxillofacial Medicine, Tehran Dental Branch, Islamic Azad University, Tehran 19395/1495, Iran; ^3^Department of Endodontics, Tehran Dental Branch, Islamic Azad University, Tehran 19395/1495, Iran

## Abstract

**Objectives:**

This study aimed to compare the antibacterial efficacy of cold atmospheric plasma (CAP), photodynamic therapy (PDT) with two photosensitizers (PSs), and diode laser for disinfection of primary mandibular second molar root canals infected with *Enterococcus faecalis* (*E. faecalis*).

**Materials and Methods:**

In this in vitro experimental study, 50 primary second primary molars underwent chemomechanical preparation of root canals. The root canals were then inoculated with *E. faecalis*. After 3 weeks of incubation, the teeth were randomly assigned to five groups of CAP, 940 nm diode laser, PDT with 445 nm laser and curcumin PS, PDT with 660 nm laser and methylene blue (MB) PS, and 2.5% sodium hypochlorite (NaOCl). Samples were collected from the vortexed root canals and cultured on agar, and the number of colonies was counted. Data were analyzed by one-way analysis of variance.

**Results:**

The percentage of reduction in bacterial count was significantly different among the study groups (*P* < 0.001). The highest reduction in bacterial count was noted in 2.5% NaOCl and the lowest in 940 nm diode laser group. The difference in bacterial count reduction between 445 nm laser + curcumin and 660 nm laser + MB (*P* = 0.989), and CAP and NaOCl (*P* = 1.000) groups was not significant.

**Conclusion:**

CAP was found to be more effective than PDT and diode laser as an adjunct to mechanical root canal disinfection of primary molars for elimination of *E. faecalis* and can serve as an alternative to 2.5% NaOCl irrigation.

## 1. Introduction

Preservation of primary teeth is imperative for harmonious growth and development of dental arch and occlusal balance in children and is a fundamental goal in pediatric dentistry [1]. Early loss of primary molars can cause malocclusion, esthetic and speech problems, and temporary or permanent functional impairment [2]. Thus, pulpectomy of primary teeth is commonly performed to prevent damage to permanent successors and early loss of primary dentition [3].

Pulpectomy is performed aiming to eliminate the microorganisms from the root canal system by mechanical debridement and chemical irrigation [4]. Success of pulpectomy depends on optimal irrigation and efficient disinfection of root canals. Preparation of curved root canals and those with anatomical variations, especially in primary molars, are among the main challenges encountered in pulpectomy [5]. Other factors such as differences in canal diameter [6], accessory foramina at the furcation site [7], isthmi, apical accessory canals, and ramifications [8] and exposure of dentinal tubules due to physiological resorption of the root can also lead to structural changes and greater permeability of the root surface to microbial toxins [9]. Such anatomical and morphological changes are more commonly seen in primary molar teeth [10].

The main cause of failure of pulpectomy in primary teeth is the residual microorganisms in the root canal system, such as *Enterococcus faecalis* (*E. faecalis*), *Streptococcus mutans*, and *Candida albicans* [11]. Of different bacterial species, *E. faecalis* is among the most resistant and refractory pathogens [12], which may remain in the root canal system after chemomechanical preparation [13] and increase the risk of endodontic treatment failure [14].

Several methods have been employed for root canal disinfection in primary teeth. Sodium hypochlorite (NaOCl) has antimicrobial and tissue-dissolving properties and is the most commonly used root canal irrigant. However, in pulpectomy of primary teeth, NaOCl can adversely affect the dental follicle of permanent successors (especially in case of presence of root resorption) and the surrounding tissues and oral mucosa [15].

Several laser types, including erbium-doped yttrium aluminum garnet (Er:YAG), neodymium-doped yttrium aluminum garnet (Nd:YAG), and diode, are available, which can efficiently disinfect the root canal system of permanent and primary teeth [16]. Also, cold atmospheric plasma (CAP) is a novel disinfection technique, which is composed of charged particles, electrons, photons, ultraviolet light, and free radicals. The constituents of CAP, such as singlet oxygen and free radicals, have antibacterial properties [17]. Antimicrobial photodynamic therapy (PDT) is another successful modality for root canal disinfection [18].

The comparative efficacy of different root canal disinfection techniques for permanent teeth has been previously investigated [19]. However, such studies are limited in primary teeth. Finding a novel technique with optimally high efficacy for root canal disinfection of primary teeth is highly important to prevent damage to permanent successors. Thus, this study aimed to compare the efficacy of CAP, PDT with two photosensitizers (PSs), and diode laser for disinfection of primary mandibular second molar root canals infected with *E. faecalis*.

## 2. Materials and Methods

This in vitro experimental study was conducted on 50 primary mandibular second molars extracted due to irreparability. The study protocol was approved by the ethics committee of Islamic Azad University (IR.IAU.TMU.REC.1401.067).

### 2.1. Sample Size

The sample size was calculated to be 10 in each group according to a study by Armand et al. [19], assuming *α* = 0.05, *β* = 0.2, mean standard deviation of log colony count to be 1.00, and effect size of 0.55 using one-way analysis of variance (ANOVA) power analysis of PASS 11. One additional specimen was added to ensure biofilm formation. Also, one additional specimen from each group did not undergo any disinfection protocol and was used to measure the intracanal colony count.

### 2.2. Eligibility Criteria

Extracted primary mandibular second molars with a minimum of two-thirds of the root remaining were collected. Teeth with physiological resorption and internal or external pathologies were excluded after radiographic examination [20]. Teeth with a history of pulpotomy or pulpectomy and perforation of pulp chamber floor were also excluded after radiographic examination [21].

### 2.3. Methodology

Debris on the tooth surface was removed by a #15 surgical scalpel (ATP, Trinon Co., Germany), and the teeth were cleaned by a disposable prophy brush (Melorin Co., China) mounted on a low-speed hand-piece (Coxo, Coxotech, China) under water coolant. The teeth were rinsed with saline and immersed in 0.5% thymol (Sigma Aldrich, Germany) for 1 week for disinfection. They were then stored in distilled water (3Sib Co., Iran) at 4°C [22].

All teeth were cut at the cementoenamel junction with a high-speed diamond disc (Crown cutter, DFS Diamond Co., Germany), and the root canals were instrumented with #15 and #20 K-files (Mani Co., Japan) followed by #25 and #30 rotary files with 4% taper (Kids File; Denco Co., China) that were used 1 mm shorter than the radiographic apex [23]. Insertion of laser fiber into the canals to 1 mm shorter than the apex was ensured. The mean length of mesiobuccal, mesiolingual, distobuccal, and distolingual canals was 10.38, 9.12, 8.88, and 8.48 mm, respectively. The root canals were irrigated with 1 mL of saline. For smear layer removal and maintaining the dentinal tubules open (for bacterial penetration), 2 mL of 17% EDTA (Asia-Shimi-Teb Co., Iran) was used in the root canals for 1 min, followed by 2 mL of 2.5% NaOCl (Nik Darman Co., Iran) for 1 min, and a final rinse with saline [24]. The apical foramen of the teeth was sealed with temporary cement (Spident Co., South Korea) to prevent apical leakage, and the external root surface was sealed with nail varnish [23].

The teeth were placed in autoclavable microtubes containing brain heart infusion broth (Merck, Germany) and autoclave-sterilized at 121°C and 15 Psi pressure for 30 min. To ensure sterilization, they were incubated at 37°C for 48 hr, and in case of turbidity of the medium, sterilization was repeated [23].

### 2.4. Microbial Culture

*E. faecalis* suspension (ATCC 29212) with 0.5 McFarland standard concentration containing 1.5 × 108 CFUs/mL was obtained from the Iranian Research Organization for Science and Technology. *E. faecalis* was cultured in broth medium and incubated at 37°C and 10% CO_2_ for 48 hr [25]. The root canals were inoculated with 15 *µ*L of *E. faecalis* suspension by a sampler for 30 s, and the teeth were incubated at 37°C and 150 rpm vibration for 3 weeks [19, 25]. Also, 15 *μ*L of sterilized brain heart infusion broth was injected into the canals on a daily basis to ensure the survival of bacteria and biofilm formation [19].

### 2.5. Scanning Electron Microscopic (SEM) Assessment

A sterile cylindrical diamond bur (Teezkavan, Iran) was used for inciso-apical sectioning of mesial and distal surfaces. The specimens were immersed in 2.5% glutaraldehyde at 4°C for 24 hr and were then dehydrated by using 70%, 85%, 90%, and 95% ethanol once and 100% ethanol twice, each for 20 min. They were then dried at room temperature. The sections were gold sputter-coated and underwent SEM assessment (CX31P, Olympus, Tokyo, Japan) to ensure biofilm formation.

### 2.6. Primary Sampling

Primary sampling was performed from one specimen in each group to measure the primary colony count. The root canals were vortexed (Vortex Mixer, KST, Iran) for 1 min at 2,500 rpm to disintegrate the biofilm. The canal contents were transferred to a 0.9 mL vial containing phosphate-buffered saline and were then cultured on agar culture medium. Following incubation at 37°C, the colonies in each plate were counted after 96 hr. The primary colony count was recorded for the purpose of comparison with the final count [26].

### 2.7. Canal Disinfection

The teeth were randomly assigned to five groups (*n* = 10) as follows, using a table of random numbers:

*Negative control:* No disinfection was performed in one specimen to ensure the sterility of the procedure and the presence of open dentinal tubules.

*Group 1: Positive control:* 2 mL of 2.5% NaOCl was used for root canal disinfection. After rinsing the root canals with NaOCl, 3 mL of 5% sodium thiosulfate (Merck, Germany) was used for 1 min to inactivate and neutralize the irrigant. A final rinse with saline was then performed [25].

**Figure 1:**
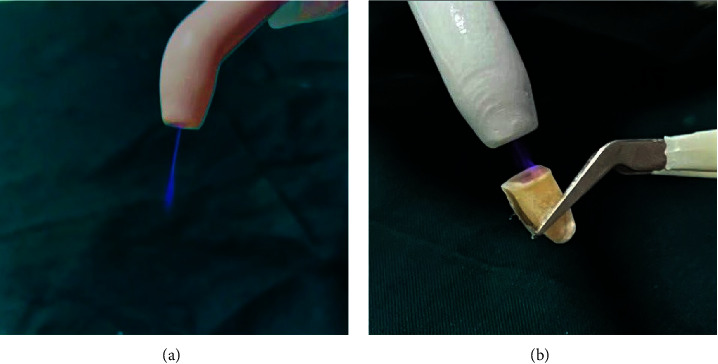
Root canal disinfection with CAP: (a) flame of device; (b) disinfection of root canals (distance between the nozzle tip and specimen is more than 5 mm in this image only for further clarification of the procedure).

*Group 2:* The teeth were subjected to CAP (Nariatech Plasmart Co., Iran) with ionized helium gas using a cold plasma hand-piece with 50 kHz frequency, 55 W input power, 3 L/min flow rate, and intensity of 4 for 60 s. The nozzle tip had 5 mm distance from the specimen surface (Figure 1) [27].

*Group 3:* Disinfection with 445 nm laser and 10.2% curcumin, which contains 40 mM curcumin in 0.05% dimethyl sulfoxide (DMSO) as PS (Adonis Gol-Darou Co., Iran); in this group, 100 *λ* of 10.2% curcumin was injected into the canals by a micropipette 120 s prior to laser irradiation [28]. Excess photosensitizer was removed by paper points. After 1 min, laser irradiation was performed. For this purpose, diode laser hand-piece (Sirona laser Co., Tehran, Iran) was used in continuous-wave mode with 445 nm wavelength and 25 mW power for 60 s [29]. The diameter of the endodontic fiber tip was 200 *µ*m. It was used 1 mm shorter than the apex. Irradiation was performed from the end of laser hand-piece tip.

*Group 4:* Disinfection with 660 nm laser and 0.02% methylene blue (MB) (Merck, Darmstadt, Germany); in this group, 100 *λ* of 0.02% MB was injected into the canals by a micropipette 120 s prior to laser irradiation [28]. Excess photosensitizer was removed by paper points. Laser irradiation was then performed. For this purpose, diode laser hand-piece (Sirona laser Co., Tehran, Iran) was used in continuous-wave mode with 660 nm wavelength and 100 mW power for 60 s [29]. Irradiation was performed from the end of laser hand-piece tip.

*Group 5: Disinfection with diode laser:* Diode laser (Epik Co., Iran) was used with 940 nm wavelength and 1W power with an endodontic fiber tip with 200 *µ*m diameter in continuous-wave mode. Irradiation was performed from the end of laser hand-piece tip. The fiber was used 1 mm shorter than the apex and moved coronally at a speed of 2 mm/s. Irradiation was performed twice, each time for 5 s (irradiation time) with a 10 s interval between the two radiation cycles [30].

After all treatments, the root canals were filled with 1 mL sterile saline [31]. Sterile paper points were utilized to remove the saline solution and bacteria (no biofilm) [19].

### 2.8. Secondary Sampling

The root canals were vortexed for 30 s to release the residual biofilm. Secondary samples were taken from the root canal contents and cultured on agar medium. The secondary colony count was measured and compared with the primary colony count in log CFUs/mL [25].

### 2.9. Statistical Analysis

Data were analyzed using SPSS version 22 by one-way ANOVA (for general comparison) and Tukey's test (for pairwise comparisons) at 0.05 level of significance.

## 3. Results

### 3.1. SEM Results

**Figure 2:**
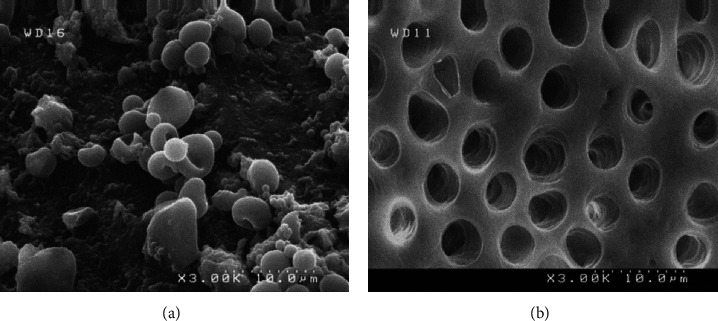
SEM micrographs: (a) specimen inoculated with *E. faecalis*; (b) dentinal tubules after elimination of smear layer (×3,000 magnification).

SEM results showed biofilm formation after 3 weeks of incubation (Figure 2(a)). The surface of dentinal tubules was completely coated with biofilm, and bacterial cells were observed.

Also, one root was considered the negative control group and did not undergo any disinfection to ensure complete sterilization of the procedures. This root was also sectioned and inspected under a SEM. The images showed open dentinal tubules and elimination of smear layer due to using 2.5% NaOCl and 17% EDTA, and no microorganism in dentinal tubules (Figure 2(b)).

### 3.2. Antibacterial Efficacy Analysis

**Figure 3:**
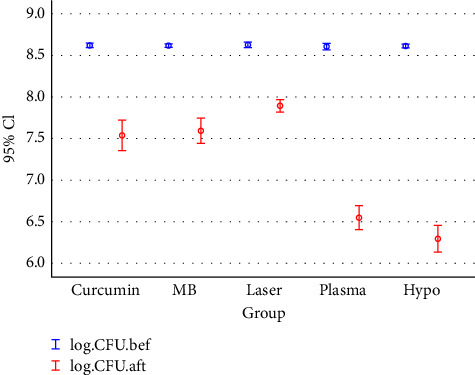
Colony count of the groups before and after the intervention (log CFUs/mL).

**Figure 4:**
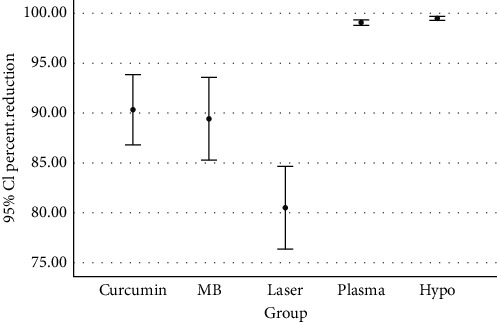
Percentage of reduction of colony count in the study groups.

Tables 1 and 2 present the colony count before and after the intervention, respectively (log CFUs/mL) (Figure 3).  Table 3 shows the percentage of reduction in colony count in each group after the intervention compared with the baseline (before the intervention) (Figure 4). The highest reduction in colony count was noted in NaOCl group (99.45%), while the lowest reduction was recorded in diode laser group (80.50%). Comparison of the five groups regarding the reduction of bacterial count by one-way ANOVA showed a significant difference among the groups in this regard (*P* < 0.001). Pairwise comparisons by Tukey's test (Table 4) showed that 445 nm laser plus curcumin was significantly more effective than diode laser for the reduction of bacterial count (*P* < 0.001). CAP was significantly more effective than 445 nm laser plus curcumin (*P* < 0.001), 660 nm laser plus MB (*P* < 0.001), and diode laser alone (*P* < 0.001) for reduction of bacterial count. Also, 2.5% NaOCl was significantly more effective than 445 nm laser plus curcumin (*P* < 0.001), 660 nm laser plus MB (*P* < 0.001), and diode laser alone (*P* < 0.001) for reduction of colony count. Moreover, 660 nm laser plus MB was significantly more effective than diode laser (*P* < 0.001) for this purpose. No other significant differences were noted (*P* > 0.05).

## 4. Discussion

This study compared the efficacy of CAP, PDT with two PSs, and diode laser for disinfection of primary mandibular second molar root canals infected with *E. faecalis*. The results showed that all the methods decreased the colony count. CAP had no significant difference with 2.5% NaOCl for the elimination of *E. faecalis* biofilm, and they were both equally effective. Considering the side effects of NaOCl in children, CAP is suggested as an alternative to NaOCl. Evidence shows that exposure of *E. faecalis* to CAP can decrease the bacterial count. Chang and Chen [32] demonstrated that CAP decreased *E. faecalis* count on glass surface after 2 min. Their results were similar to the present findings, although they cultured *E. faecalis* on glass and did not use mature biofilm. Rupf et al. [33] evaluated the antibacterial effects of CAP on Gram-positive and Gram-negative species and showed that its antibacterial effects on microorganisms cultured on tooth surfaces were smaller than its effects on planktonic bacteria or biofilm cultured on agar plate. This finding can be due to dentin surface porosity and biofilm penetration, which would prevent exposure of bacteria to antibacterial agents, laser, and plasma.

A previous study showed the optimal efficacy of CAP against *E. faecalis* biofilm on the root surface of permanent teeth. However, since the density of dentinal tubules of primary teeth is higher than permanent teeth, it was expected that greater biofilm penetration and their inaccessibility for CAP would result in lower efficacy of CAP in primary teeth compared with permanent teeth [34]. This study was the first to assess the effect of CAP for root canal disinfection in primary teeth and reported positive results.

Schaudinn et al. [35] showed that although CAP significantly decreased the root canal bacterial count, its efficacy was significantly lower than that of 6% NaOCl. Difference between the present results and those of Schaudinn et al. [35] can be due to the fact that NaOCl remained in the canal for 30 min in their study, which is obviously not feasible in the clinical setting.

The disinfecting efficacy of CAP depends on its duration of use [19]. Chang and Chen [32] found that the antimicrobial effects of CAP were time-dependent and 3 min of exposure yielded the best results. Armand et al. [19] reported a significant reduction of biofilm microorganisms due to exposure to helium or He/O_2_ CAP for 4, 6, and 8 min. In this study, considering the significance of fast conduction of procedures in pediatric dentistry, CAP was used for 60 s and caused about 99% reduction in colony count after 1 min. Such an acceptable result in primary root canal disinfection in a short time is highly valuable for application in the clinical setting.

Armand et al. [19] reported similar antibacterial efficacy of helium CAP and PDT with MB; however, in the present study, the antibacterial efficacy of helium CAP was higher than PDT. Also, it should be noted that 3-week biofilm was used in the present study, while 1-week biofilm was used by Armand et al. [19]. One-week biofilm cannot perfectly simulate the clinical setting [36]. Significantly higher antibacterial efficacy of CAP compared with PDT can be due to the gas nature of plasma since gas can directly penetrate into the root canal porosities and interact with the biofilm and eliminate it.

Yao et al. [37] found no significant difference in colony count reduction following CAP and 2% chlorhexidine for 10 min, and CAP decreased 1-day and 3-week *E. faecalis* colony count of biofilm with no adverse effect on dentin structure and no temperature rise. Their results were consistent with the present findings, although they did not well simulate the clinical setting.

In the present study, PDT with two PSs of curcumin and MB was evaluated, and the results revealed no significant difference between the efficacy of PDT with 445 nm laser and curcumin and 660 nm laser and MB. The same results were reported by Monzavi et al. [38]. Antibacterial effects of PDT are related to its photochemical and photothermal effects, while CAP causes no thermal damage to dentin, which is a great advantage, and therefore, CAP is a better option for root canal disinfection of primary teeth due to the presence of dental follicle of permanent successors.

In the present study, the antimicrobial efficacy of PDT was significantly inferior to irrigation with NaOCl in the reduction of biofilm in primary root canals. The same result was reported in previous studies. Miere et al. [39] reported that the disinfecting efficacy of PDT with toluidine blue and 100 mW laser power was significantly lower than that of 2.5% NaOCl. Nonetheless, they used 48 hr biofilm of *E. faecalis*, which was a drawback.

Öter et al. [31] assessed the efficacy of PDT with 670 nm laser and tolonium chloride as PS against a 1-week biofilm of *E. faecalis* in primary root canals; they showed lower efficacy of this modality compared with 2.5% NaOCl, which was in agreement with present findings. However, another study by Martin et al. [20] indicated that PDT with 660 nm laser and toluidine blue caused a greater reduction in *E. faecalis* count compared with chemomechanical preparation alone. Pinheiro et al. [40] found that chemomechanical root canal preparation alone decreased intracanal biofilm in necrotic primary molars by 82% while using PDT as an adjunct resulted in 98% bacterial reduction, which was different from the percentage of reduction obtained in the present study. Difference between the results of clinical and in vitro studies regarding the efficacy of PDT with curcumin or MB can be due to the fact that in clinical studies, samples are collected by a paper point from the canal, which only collects bacteria on dentin surface, and cannot access the bacteria penetrated into dentinal tubules; whereas, the root canals were vortexed in the present study and then the samples were collected. Thus, the biofilm in dentin depth was also collected. The present results, therefore, appear to better simulate the oral environment.

The present results also showed lower efficacy of 940 nm diode laser than PDT with MB or curcumin. Diode laser had the lowest disinfecting efficacy against 3-week biofilm of *E. faecalis*.

In line with the present results, Attiguppe et al. [41] showed that PDT was significantly more effective than diode laser in the reduction of 4-week biofilm of *E. faecalis* in primary root canals. They used 810 nm diode laser with 1.5 W power for 60 s along with indocyanine green as PS for PDT. Use of PS results in greater energy accumulation and better photothermal effect, which leads to the degradation of bacterial cell wall and cell death.

Number of studies on the disinfecting efficacy of diode laser in primary root canals is limited, and the majority of available studies have been conducted on permanent teeth. Kuvvetli et al. [42] demonstrated that 810 nm diode laser with 300 mW power had an antibacterial effect comparable to that of 5.25% NaOCl in the elimination of 24 hr biofilm of primary molars. Difference between their results and the present findings can be due to the degree of maturity of bacterial biofilm, which was higher in the present study, and is an advantage of the current investigation.

Dai et al. [43] evaluated the disinfecting effect of 810 nm diode laser in comparison with NaOCl irrigation on 3-week biofilm of *E. faecalis* in primary molars. In contrast to the present results, they found that diode laser eliminated almost 96% of the primary root canal bacteria, and its efficacy was significantly higher than that of NaOCl. Difference between their results and the present findings may be due to the use of diode laser with 1 W power in the present study and 2 W power in their study. The antibacterial effect of diode laser is due to the generated heat; thus, using a higher power increases its efficacy. Despite the higher antibacterial efficacy of diode laser with higher powers, lower powers are recommended for primary teeth in the clinical setting because the primary teeth have a thinner dentinal wall, which results in greater temperature rise in the external root surface that may damage the permanent tooth bud. Bahrololoomi et al. [30] reported that using 1.5 W diode laser effectively decreased *E. faecalis* count without damaging the periodontal structures.

In vitro design was a limitation of this study since a number of influential factors, such as periapical response, cooperation of pediatric patients, and many other tooth- and tissue-related parameters, cannot be well simulated in vitro. Thus, the generalization of results to the clinical setting should be done with caution. Future studies with different CAP parameters and in vivo studies are required to assess its intraoral effects.

## 5. Conclusion

The present results showed that CAP was more effective than PDT and diode laser as an adjunct to mechanical root canal disinfection of primary molars for elimination of *E. faecalis* and can serve as an alternative to 2.5% NaOCl irrigation.

## Figures and Tables

**Table 1:** Colony count before the intervention (log CFUs/mL) (*n* = 10).

				95% Confidence interval for mean		Minimum	Maximum
				Lower bound	Upper bound		
Laser + curcumin	8.61	0.04	0.01	8.58	8.64	8.56	8.69
Laser + MB	8.61	0.02	0.008	8.59	8.63	8.58	8.65
Diode laser	8.62	0.04	0.01	8.59	8.65	8.56	8.70
Cold plasma	8.60	0.05	0.01	8.56	8.64	8.55	8.68
Hypochlorite	8.60	0.02	0.008	8.58	8.62	8.58	8.68
Total	8.61	0.03	0.005	8.60	8.62	8.55	8.70

**Table 2:** Colony count after the intervention (log CFUs/mL) (*n* = 10).

	Mean	Std. deviation	Std. error	95% Confidence interval for mean		Minimum	Maximum
				Lower bound	Upper bound		
Laser + curcumin	7.53	0.25	0.08	7.35	7.72	7.00	7.85
Laser + MB	7.59	0.21	0.06	7.43	7.74	7.30	7.99
Diode laser	7.89	0.10	0.03	7.82	7.96	7.71	7.99
Cold plasma	6.54	0.20	0.06	6.40	6.69	6.30	6.85
Hypochlorite	6.29	0.22	0.07	6.13	6.45	6.00	6.60
Total	7.17	0.66	0.09	6.98	7.36	6.00	7.99

**Table 3:** Percentage of reduction in colony count in each group after the intervention compared with baseline (*n* = 10).

Group	Mean	Std. deviation	Std. error	95% Confidence interval for mean		Minimum	Maximum
				Lower bound	Upper bound		
Laser + curcumin	90.32	4.93	1.56	86.78	93.85	81.56	97.73
Laser + methylene blue	89.40	5.77	1.82	85.28	93.53	76.27	95.06
Diode laser	80.50	5.74	1.81	76.39	84.61	73.30	89.86
CAP	99.04	0.38	0.12	98.77	99.32	98.54	99.58
NaOCl	99.45	0.25	0.08	99.27	99.64	99.04	99.76
Total	91.74	8.18	1.15	89.42	94.07	73.30	99.76

**Table 4:** Pairwise comparisons of the groups regarding the percentage of reduction in colony count using Tukey's test.

Group (*I*)	Group (*J*)	Mean difference (*I* − *J*)	Std. error	Sig.	95% Confidence interval	
					Lower bound	Upper bound
Laser + curcumin	Laser + MB	0.91	1.90	0.989	−4.50	6.33
	Diode laser	9.81^*∗*^	1.90	<0.001	4.39	15.23
	Cold plasma	−8.72^*∗*^	1.90	<0.001	−14.14	−3.30
	Hypochlorite	−9.13^*∗*^	1.90	<0.001	−14.55	−3.71

Laser + MB	Curcumin	−0.91	1.90	0.98	−6.33	4.50
	Diode laser	8.90^*∗*^	1.90	<0.001	3.48	14.32
	Cold plasma	−9.63^*∗*^	1.90	<0.001	−15.05	−4.21
	Hypochlorite	−10.04^*∗*^	1.90	<0.001	−15.46	−4.62

Diode laser	Curcumin	−9.81^*∗*^	1.90	<0.001	−15.23	−4.39
	Laser + MB	−8.90^*∗*^	1.90	<0.001	−14.32	−3.48
	Cold plasma	−18.54^*∗*^	1.90	<0.001	−23.95	−13.12
	Hypochlorite	−18.95^*∗*^	1.90	<0.001	−24.37	−13.53

Cold plasma	Curcumin	8.72^*∗*^	1.90	<0.001	3.30	14.14
	Laser + MB	9.63^*∗*^	1.90	<0.001	4.21	15.05
	Diode laser	18.54^*∗*^	1.90	<0.001	13.12	23.95
	Hypochlorite	−0.41	1.90	1.00	−5.83	5.00

Hypochlorite	Curcumin	9.13^*∗*^	1.90	<0.001	3.71	14.55
	Laser + MB	10.04^*∗*^	1.90	<0.001	4.62	15.46
	Diode laser	18.95^*∗*^	1.90	<0.001	13.53	24.37
	Cold plasma	0.41	1.90	1.00	−5.00	5.83

^*∗*^The mean difference is significant at the 0.05 level.

## Data Availability

The data used to support the findings of this study were supplied by the corresponding author under license, and data will be available on request. Requests for access to these data should be made to the corresponding author.
